# Progress, highlights and perspectives on NiO in perovskite photovoltaics

**DOI:** 10.1039/d0sc02859b

**Published:** 2020-07-13

**Authors:** Diego Di Girolamo, Francesco Di Giacomo, Fabio Matteocci, Andrea Giacomo Marrani, Danilo Dini, Antonio Abate

**Affiliations:** Department of Chemical, Materials and Production Engineering. University of Naples Federico II Pzz.le Vincenzo Tecchio 80 Naples 80125 Italy diego.digirolamo91@gmail.com; Department of Chemistry, University of Rome La Sapienza Pzz.le Aldo Moro 5 Rome 00185 Italy; C.H.O.S.E.- Center for Hybrid and Organic Solar Energy, Department of Electrical Engineering, University of Rome Tor Vergata Via del Politecnico 1 00133 Rome Italy; Institute for Silicon Photovoltaics, Hemlholtz Zentrum Berlin Kekulestraße 5 D-12489 Berlin Germany antonio.abate@helmholtz-berlin.de

## Abstract

The power conversion efficiency (PCE) of NiO based perovskite solar cells has recently hit a record 22.1% with a hybrid organic–inorganic perovskite composition and a PCE above 15% in a fully inorganic configuration was achieved. Moreover, NiO processing is a mature technology, with different industrially attractive processes demonstrated in the last few years. These considerations, along with the excellent stabilities reported, clearly point towards NiO as the most efficient inorganic hole selective layer for lead halide perovskite photovoltaics, which is the topic of this review. NiO optoelectronics is discussed by analysing the different doping mechanisms, with a focus on the case of alkaline and transition metal cation dopants. Doping allows tuning the conductivity and the energy levels of NiO, improving the overall performance and adapting the material to a variety of perovskite compositions. Furthermore, we summarise the main investigations on the NiO/perovskite interface stability. In fact, the surface of NiO is commonly oxidised and reactive with perovskite, also under the effect of light, thermal and electrical stress. Interface engineering strategies should be considered aiming at long term stability and the highest efficiency. Finally, we present the main achievements in flexible, fully printed and lead-free perovskite photovoltaics which employ NiO as a layer and provide our perspective to accelerate the improvement of these technologies. Overall, we show that adequately doped and passivated NiO might be an ideal hole selective layer in every possible application of perovskite solar cells.

## Abstract 2

The power conversion efficiency of NiO based perovskite solar cells has recently hit a record 22.1%. Here, the main advances are reviewed and the role of NiO in the next breakthroughs is discussed.

## Graphical abstract

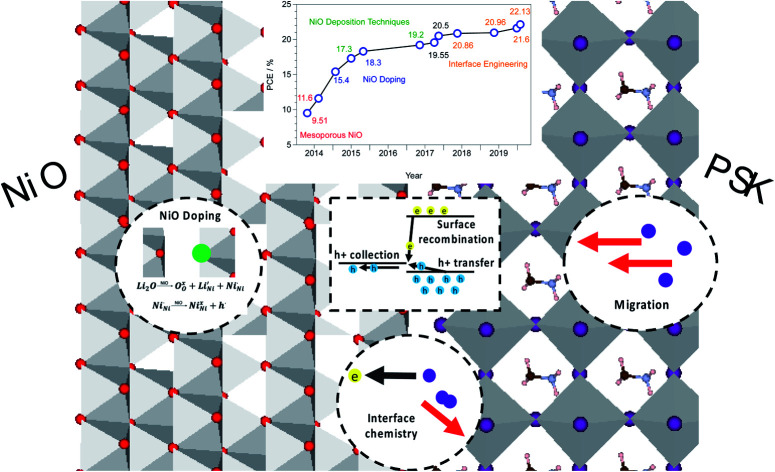

## 1. Introduction

Metal halide perovskite solar cells (PSCs) are based on a p–i–n junction, with the perovskite absorber sandwiched between n-type and p-type semiconductors acting as selective layers.^1–3^ A steep and tunable light absorption onset, high ambipolar photoconductivity and long photocarrier lifetimes are the key properties of lead halide perovskites behind the rise in power conversion efficiency (PCE) to above 25%.^4^ To make the most of perovskite optoelectronics, selective layers have to extract the photocurrent without introducing ohmic losses and energetic barriers and have to minimise the non-radiative recombination at the interfaces.^5–8^ SnO_2_,^9,10^ TiO_2_ (ref. 11 and 12) and fullerenes^13,14^ are the best candidates for electron selective layers (ESLs). On the other hand, although materials with excellent performances in terms of efficiency and stability have been developed,^15–17^ Spiro-OMeTAD and PTAA remain the standard hole selective layers (HSLs).

Spiro-OMeTAD films have a very low conductivity (below 10^−7^ S cm^−1^), and ingenious doping strategies have been developed to overcome this limitation and minimise ohmic losses. LiTFSI is employed to catalyse Spiro-OMeTAD oxidation from atmospheric oxygen, resulting in a 100-fold increase in conductivity.^18^ Alternatively, protic ionic liquids^19^ and molecules with high electron affinity can act as dopants, as in the case of F4TCNQ doped PTAA.^20^ Besides the HSL conductivity, the interplay between charge transfer and charge recombination at the perovskite/layer interface is mostly affected by interface energetics and defect density. The highest occupied molecular orbital (HOMO) of PTAA (−5.2 eV) and Spiro-OMeTAD (−5.1 eV) lies slightly above the valence band (VB) of CH_3_NH_3_PbI_3_.^21^ This induces a driving force for hole extraction and ensures a high built-in potential, which slows down the non-radiative recombination.^5^ Additionally, a passivation layer^7^ or careful molecular tailoring^22,23^ can be exploited to minimise the interface recombination.

NiO has a VB within −5.0 eV and −5.4 eV which leads to a good energy alignment with common lead halide perovskites. Its main drawback is the relatively low open circuit voltage (*V*_oc_) of devices, due to high interface recombination. Nonetheless, NiO is the most promising inorganic p-type hole selective layer for perovskite photovoltaics, showing the highest efficiencies and better reproducibility among different research groups when compared to other suitable inorganic p-type semiconductors.^24–26^ Compared to Spiro-OMeTAD and PTAA and other organic hole selective layers, NiO is orders of magnitude less expensive^27^ and holds great promise in terms of PSCs' stability. The solar cells' failure commonly follows the intermixing and reaction between perovskite and adjacent layers, especially metal electrodes. Molecular or polymeric selective layers hardly prevent this interdiffusion.^28–32^ In addition to that, dopants such as LiTFSI and *tert*-butyl pyridine can diffuse and react with the perovskite and layers,^33–35^ further degrading the device performances. A uniform NiO layer ideally insulates the perovskite film from the electrode, thus preserving the structural integrity of the device.

In this review, we highlight the main advancement concerning the implementation of NiO in perovskite solar cells, a successful story yielding a PCE of over 22% and operational stabilities of over 1000 h. While not aiming at a complete coverage of the latest research on NiO (for which we recommend ref. 25 and 26), we focus on the material chemistry behind the NiO working principles to understand the effect of NiO defects, doping density and surface chemistry on the perovskite solar cell behaviour. We discuss industrially attractive NiO implementation by considering scale-up compatible processes and flexible photovoltaics. Finally, we summarise the latest findings and provide guidelines for the future development of printable and lead-free perovskite solar cells, the next breakthroughs in perovskite photovoltaics.

## 2. NiO in perovskite solar cells

**Fig. 1:**
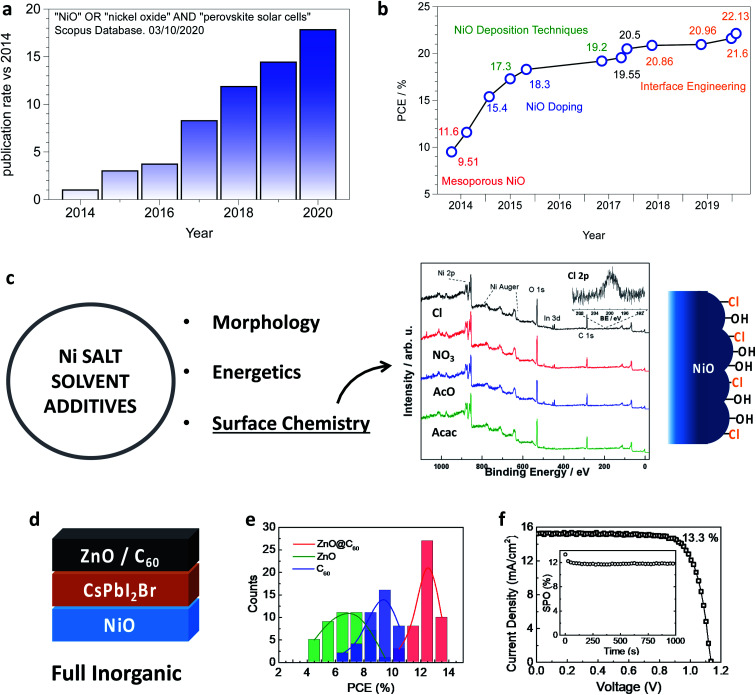
(a) Publication rate evaluated as the number of papers per month as calculated using the Scopus database for the “NiO” and “perovskite solar cells” keywords. (b) Record PCE chart for p–i–n perovskite solar cells employing NiO as a hole selective layer. Ref. 36–46: the key approaches leading to record efficiency are indicated. (c) Scheme linking the NiO precursor formulation to the critical properties of the final NiO films. The effect of the Ni precursor on the surface chemistry is highlighted by XPS investigation (as described in ref. 47) on NiO films processed from NiCl_2_, Ni(NO_3_)_2_, Ni(AcO)_2_ and Ni(acac)_2_ showing that chloride remains bound to the surface. (d) Example of full inorganic (perovskite and selective layers) PSC configuration. (e) Dependance of the PCE of full inorganic NiO/CsPbI_2_Br PSCs on the electron selective layer. (f) The best cell from Fig. 1d with the complete inorganic configuration NiO/CsPbI_2_Br/ZnO@C_60_. (e) and (f) are reprinted with permission from ref. 48. Copyright 2018 American Chemical Society.

Nickel oxide is the most attractive inorganic p-type semiconductor to act as a hole selective layer in perovskite photovoltaics, with the research community being increasingly interested in this as shown in Fig. 1a, where the “publication rate” (*i.e.* number of papers per month) increases by a factor 20 in about five years. NiO is usually implemented in an inverted (p–i–n) architecture, with the perovskite film grown on top of NiO from which light shines through the device. In this configuration, efficiencies above 20% have been demonstrated. Moreover, tremendous efforts resulted in a vast range of alternative NiO deposition techniques, including industrially attractive routes. Some of these techniques are, in principle, compatible with processing NiO on top of a perovskite film, which would enable full-inorganic n–i–p perovskite solar cells, an ideal solution to combine the highest efficiency and stability.

### 2.1 P–i–n perovskite solar cells

The power conversion efficiency justifies the prominent role of NiO as an inorganic hole selective layer, with several demonstrations above 20% in the last two years and a (not certified) record of 22.13%.^38^ In Fig. 1b we show the record chart for single-junction perovskite solar cells employing NiO as the hole selective layer, which also highlights the most critical approaches developed to effectively implement NiO into perovskite solar cells, which will be discussed throughout this review article. The most efficient NiO based PSC has a planar p–i–n architecture, where a ∼20 nm thin NiO layer is deposited employing sol–gel chemistry principles or from a NiO nanoparticle ink. One of the most robust protocols consists of the spin-coating of a nickel acetate solution in 2-methoxyethanol with monoethanolamine as the stabilising/complexing additive.^49^ Alternatively, by adding acetylacetonate to a nickel nitrate solution a combustion reaction allows reducing the annealing temperature from above 300 °C to 250 °C,^50^ and even to 150 °C when copper as a dopant is included.^51^ In our lab, we achieved 20% PCE (unpublished results) by employing a nickel chloride solution with nitric acid as the additive. Notably, our procedure spontaneously leads to a chloride capped NiO film, as shown in Fig. 1c, which is particularly interesting since the chloride functionalisation was found beneficial in terms of interface recombination for TiO_2_ (ref. 52) and SnO_2_.^53^ Finally, the spin-coating of NiO nanoparticles ink brings about the main advantage of the abatement of post-deposition annealing.^54,55^ We remark that the selection of the best performing “NiO recipe” is not an easy task. In fact, a meaningful comparison between the different procedures will be possible only with a general improvement in the description of the experimental methods^56^ (which will also positively impact Fig. 1b). As an example, the relative humidity is difficult to control and almost never reported, but it has a strong influence on the formation of metal oxides, where water may act as a reagent.

The highest efficiency NiO-based PSCs exploit formamidinium based mixed-cation mixed-halide composition, closely following the general trend in perovskite photovoltaics. However, NiO might also be particularly well suited for the fabrication of fully inorganic perovskite solar cells. Avoiding the organic A-site cation improves the thermal^57^ and environmental^58,59^ stability of APbX_3_ perovskites. Furthermore, the introduction of inorganic semiconductors at both layers inherently encapsulate the perovskite. Typically, a CsPbI_2_Br stoichiometry (or even richer in bromide^57^) is adopted to overcome the phase instability of CsPbI_3_, stable at room temperature in the non-photoactive δ-CsPbI_3_ phase (even if it is possible to substantially extend the metastability of the CsPbI_3_ perovskite phase in Cs-rich condition^60^ or by tuning the strain of the thin film^61^). The adoption of the composite ZnO@C_60_ electron selective layer in NiO/CsPbI_2_Br based PSCs was found crucial to achieve an efficiency above 13% in full inorganic perovskite solar cells (Fig. 1c and d),^48^ lately improved by doping C_60_ with LiClO_4_ and tris(pentafluorophenyl)borane (TPFPB) to attain a remarkable 15% PCE.^62^ Further improvement will likely be achieved by treating the NiO surface, as shown by Yang *et al.* to boost the efficiency of NiO/CsPbBr_2_I based PSCs from 6.3% to 9.5%.^63^

### 2.2 Beyond spin-coating

The up-scaling process became crucial for the exploitation of perovskite solar cell technology at the industrial level. Recently, research institutes and R&D companies have made considerable efforts to speed up the manufacturing process of perovskite solar modules at a high technology readiness level (TRL). With this aim, the research on cost-effective, scalable, and high throughput deposition techniques needs to be addressed as a hot topic for the future development of PSC photovoltaic technology. Considering the whole manufacturing process, necessary actions have to be considered concerning the scalable deposition of the entire stack forming PSC devices. In this topic, the uniform and scalable deposition of hole transport layers plays an essential role in both n–i–p and p–i–n device architectures. On the other hand, the processing temperature and operational stability of HSLs have to be taken into account too. NiO offers considerable opportunities to match all these requirements.

NiO layers have been deposited by well-established industrially relevant physical and chemical vapour deposition techniques. Techniques like sputtering,^64,65^ pulsed laser deposition (PLD),^66^ thermal^67^ and electron-beam evaporation,^68^ chemical vapour deposition (CVD) and atomic layer deposition (ALD)^69^ have been introduced for the manufacturing of NiO layers for highly efficient inverted p–i–n perovskite solar cells, approaching 20% PCE.^68^ Whether these techniques could represent the convenient industrial process for NiO deposition would depend, besides the quality of the NiO layer, on the cost and throughput. Ideally, the use of vacuum-free printing techniques based on solution processing might fit those requirements. Methods like chemical bath deposition (CBD),^70^ electrodeposition,^42,71^ spray pyrolysis,^72,73^ blade coating and screen-printing are, therefore, highly attractive. At the same time, it could be convenient to adopt processing routes which can be straightforwardly implemented into the already operative industrial line. With this in mind, an available and cost-effective solution could consist of the deposition of a NiO film during the manufacturing process of transparent conductive oxides (TCOs). The most commonly used TCOs for PSC technology are indium tin oxide (ITO) and fluorine-doped tin oxide (FTO). The ITO coating is generally deposited by sputtering and commercially available on both rigid glass and flexible plastic substrates. FTO is made by spray pyrolysis deposition (SPD) at a high processing temperature limiting its use on glass substrates only. Sputtering and SPD are both suitable techniques for obtaining a high-quality NiO film, which makes the integration of the deposition in the manufacturing process of TCOs the most advantageous option. Sequential manufacturing of the TCO and the NiO HSL in the same production line can guarantee better reproducibility and a high throughput process. Different is the case of tandem photovoltaics, where the PSC acts as a wide-bandgap device in combination with silicon or CIGS. Mechanically stacking the PCS on top of the silicon solar cell, as shown by Lamanna *et al.*,^74^ would directly enable every NiO deposition demonstrated in the literature. Nonetheless, aiming at processing the PSC on top of a textured silicon solar cell in a monolithic tandem device, sputtering might be an ideal technique for the deposition of a 15–20 nm thick conformal NiO layer, as demonstrated by Hou *et al.* who achieved a certified PCE of 25.7%.^75^ Alternatively, Jost *et al.* employed ALD to deposit NiO on top of a rough CIGSe solar cell, and achieved a 21.6% PCE when introducing a thin PTAA passivation layer to improve the *V*_oc_ and fill factor (FF) of the perovskite device.^76^

### 2.3 N–i–p perovskite solar cells

Specular to the approach of full inorganic p–i–n perovskite solar cells, the introduction of NiO as a hole selective layer into an n–i–p architecture is one of the most promising routes to stabilise perovskite photovoltaics. Excellent demonstrations are the CuSCN/rGO hole selective layer developed by Arora *et al.*,^77^ and the 16 months of shelf-life stability by employing NiO and TiO_2_ as layers in a p–i–n architecture demonstrated by Zhao *et al.*^78^

Despite the great promise, few demonstrations of NiO in n–i–p PSCs have been reported to date, all exploiting inks of NiO nanoparticles (NPs) based on solvents orthogonal to perovskite due to the high temperature needed for the other deposition of NiO. The standard approach is to produce functionalised NiO nanoparticles, capped with an organic species which allows solubility in aromatic or alcoholic solvents.^79–82^ The PCE is within 12% and 9%, due to the negative impact of the organic shell on the conductivity of the NiO layers and on the hole transfer from the perovskite. In fact, Liu *et al.* observed an increase in PCE in the p–i–n architecture after removing part of the ligands with an UV-ozone treatment,^81^ a procedure not compatible with n–i–p devices. Another reason for the relatively poor performances might be the quality of the NiO film, in terms of NP packing and electrical layer with the perovskite. To this end, a finer tuning of the deposition technique, NP size and size dispersity might enable more efficient layers. An alternative is to fill the NiO NP film with an organic or polymeric hole selective layer, as in the case of the NiO|Spiro-OMeTAD bi-layer shown by Li *et al.*,^83^ who demonstrated a 21.6% efficiency. Moreover, the authors suggest that NiO might act as a protecting layer, slowing down the diffusion of Spiro-OMeTAD dopants into the perovskite layer, thus stabilising the device.

### 2.4 Safety hazards

The use of NiO presents some safety concerns due to its toxicity. Nickel itself is suspected of causing cancer, can cause an allergic reaction and may cause damage to organs. The NIOSH suggests a recommended exposure limit of 0.015 mg m^−3^ for continuous exposure of 8 hours.^84^ Nickel oxide shows similar hazards; however, the potential exposure when deposited as a well-bonded thin film can be considered very limited. The main risk occurs in the deposition phase, raising safety concerns for the operators. In a sol–gel synthesis, each nickel precursor has specific toxicity: while nickel acetate is less harmful than nickel, nickel nitrate and nickel chloride present some additional risks.^27^ Commonly used solvents like 2-methoxy ethanol, acetylacetone, and ethylene glycol are also increasing the risk if the operator is exposed to the sol–gel ink or its fumes.

Nevertheless, with the appropriate use of personal protective equipment (PPE) as well as a confined deposition environment, it is possible to minimise risks. Other solvents such as alcohols or water can lower the requirements for ventilation, and their use should be encouraged. When nanoparticles are used, their smaller size and their airborne nature represent a significant increase in the risks. Recent studies suggested that the exposure limit should be 10 times lower than bulk nickel oxide,^85^ and it is essential to have strict control on the handling of dry nanopowders. For the same reason it is vital to ensure good adhesion with the substrate (especially on a flexible substrate in a roll-to-roll production) to avoid the release of airborne particles. When NiO is deposited by sputtering or e-beam evaporation, the film is usually well bonded to the substrate and the risks are mostly limited to the periodic cleaning of the chamber or due to deposition of flakes from the target on samples. We want to emphasise that it is essential to develop appropriate safety procedures when handling this material, both during the research and the development phase as well as in the eventual industrialisation phase. We also believe that with good control of the thin film deposition procedure, it is possible to use Ni and NiO without risks, as the material is already widely used in the industry.

## 3. Doping of NiO

NiO is an insulator in its stoichiometric form, with its p-type conductivity arising from the self-doping mechanism due to the Ni^2+^ vacancy (
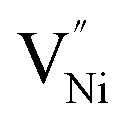
, the thermodynamically most abundant point defect). In Kröger–Vink notation the self-doping mechanism can be written using eqn (1) and (2):1
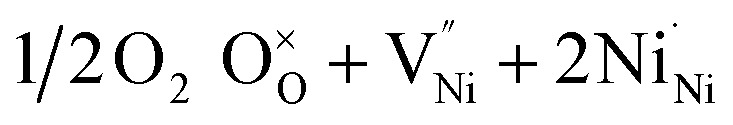
2
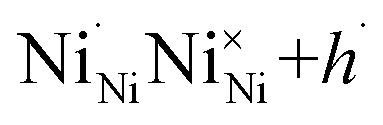


The twofold negative charge of the nickel vacancy is compensated for by the stabilisation of the Ni^3+^ oxidation state 
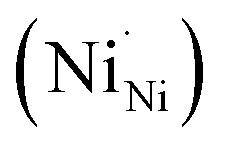
, which acts as an acceptor shallow level. This picture serves as a basis to understand the NiO doping, which could proceed through the substitutional replacement of Ni^2+^ with a cation with a lower oxidation state (*e.g.* Li^+^ or Ag^+^) or by modifying the oxidation state (non-stoichiometry) of the film.^86^ Moreover, dopants can influence the optoelectronics of NiO by modifying its work function or ionisation potential, as in the case of alkaline-earth cation doping.^64,87^ Before discussing in detail the doping mechanisms from alkaline and transition metal cations it is important to remark that non-metal and molecular doping is also suitable to improve the NiO performances. Zhou *et al.*^88^ reported the improvement of hole extraction from perovskite by including nitrogen (through guanidinium nitrate) in NiO. Additionally, some of the highest performances (>20% PCE) have been demonstrated by implementing molecular doping either confined at the surface^45^ or throughout the whole NiO layer (in films deposited from NiO nanoparticle ink).^38^ The molecules employed (such as F6TCNNQ) extract electrons from NiO in virtue of their high electron affinity (above 5.3 eV) and establish a strong interaction with the NiO surface, which ensures the stability of the molecular doping after the processing of perovskite. Notably, the surface doping was demonstrated by employing electrostatic force microscopy and distinguished from the mechanism of surface passivation.^45^

### 3.1 Alkali cation doping

In Kröger–Vink notation, considering Li^+^, the alkaline cation doping of NiO *via* Ni^2+^ substitutional replacement^94^ can be rationalised with eqn (3) and (4):3
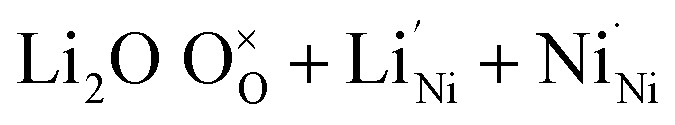
4
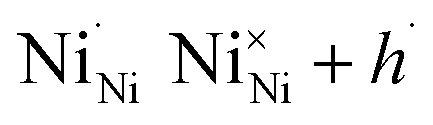


**Fig. 2:**
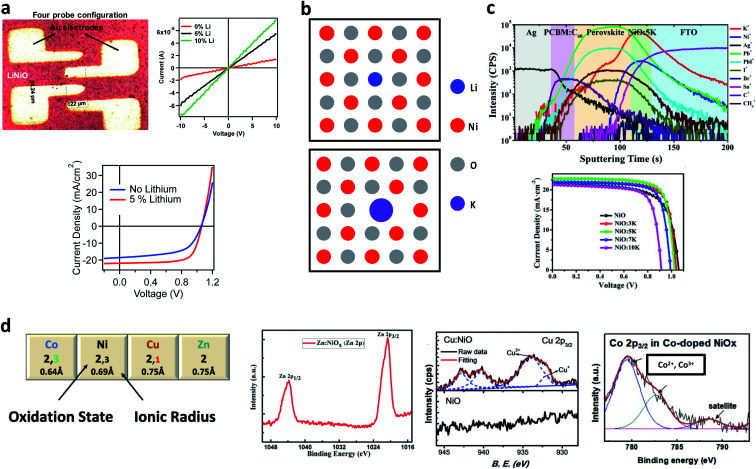
(a) Configuration for measuring the conductivity of Li : NiO with the improvement of *J*_sc_ and FF in the perovskite solar cell. Reproduced with permission from ref. 89. Copyright 2018 John Wiley and Sons. (b) Scheme for the substitutional replacement of Ni^2+^ with small Li^+^ and large K^+^. (c) Depth profiling of a perovskite solar cell showing that K^+^ is also found in the perovskite after diffusion from K : NiO. Below are the *J*–*V* curve at different doping levels for K : NiO. Reproduced with permission from ref. 90. Copyright 2019 Royal Society of Chemistry. (d) The most abundant oxidation state with the most common ionic radius for Ni and appropriate transition metal dopants. XPS spectra for doped NiO films are reported to highlight the mixture of oxidation states in the case of Cu and Co. Reproduced with permission from ref. 91. Copyright 2018 American Chemical Society. Reproduced with permission from ref. 92. Copyright 2018 John Wiley and Sons. Reproduced with permission from ref. 93. Copyright 2019 Elsevier.

The negative charge of 
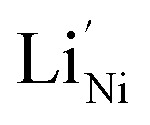
 is compensated for by the Ni^3+^ acceptor level. A significant increase in the FF and *J*_sc_ of PSCs follows the enhanced conductivity of NiO upon lithium doping, as shown in Fig. 2a and reported by many authors.^66,89,95,96^ Moving down the alkaline group, the energetic cost for Ni^2+^ substitutional replacement increases due to ionic radius mismatch (*e.g.* Ni^2+^ = 0.69 Å, Li^+^ = 0.76 Å, and Cs^+^ = 1.67 Å), and different doping mechanisms. Interestingly, a combined XAFS and theoretical investigation on molecular beam epitaxy (MBE) deposited K-doped NiO films suggested that potassium tends to cluster and float to the NiO surface. At the same time the formation in traces of 
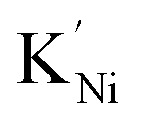
 is promoted by additional adjacent oxygen and/or nickel vacancies in order to compensate for the large radius of K^+^,^97^ as sketched in Fig. 2b. This is coherent with the work from Yin *et al.* reporting an increase in PCE from 15.77% to 18.05% by doping K^+^ into NiO.^90^ In fact, along with an increased conductivity for the K : NiO hole selective layer, a sizable amount of potassium was found to diffuse out of the NiO crystallites and inside the perovskite film (Fig. 2c). Similarly, in our work, we found little if any evidence for Na^+^ inclusion into the NiO lattice. However, the sodium segregation out of the NiO crystallites was beneficial in terms of trap passivation.^98^ The segregation of alkaline cations at the NiO surface might improve the device performances thanks to the beneficial interaction with the perovskite, as observed for accidentally included sodium^99^ and deliberately included potassium.^100,101^ It must be considered that the spatial distribution of the dopants might vary with time, thus modifying the performances of the solar cell. In particular, the small size of lithium makes this cation mobile in perovskite^35^ and NiO.^102^ Therefore, a sizeable lithium drift might be expected when the photovoltage develops at the NiO/perovskite interface. Larger dopants could be exempt from this criticality. With this in mind, the increase in conductivity for Cs-doped NiO is particularly interesting, which in synergy with an increased work function (from 4.89 eV to 5.11 eV) leads to the rise of the PCE from 16% to above 19%.^103^ Notably, Kim *et al.* observed a decrease in the metallic Ni content by introducing Cs^+^ into the precursor solution,^104^ unveiling an unpredictable role in enhancing the phase purity of solution-processed NiO for the alkaline additive.

### 3.2 Transition metal cation doping

Silver is the only transition metal (TM) cation employed as a dopant for NiO stable in the +1 oxidation state, thus bringing about a doping mechanism similar to that of lithium.^105^ Interestingly, a synergy between lithium and silver in tailoring the optoelectronic properties of NiO has been reported by Xia *et al.*,^96^ who demonstrated a 19.24% efficient PSC.

To understand the doping effect of other TM cations, we have to consider their respective stable oxidation states, as shown in Fig. 2d. Zn^2+^ is isovalent with Ni^2+^, and the ionic radius mismatch is within 10%, both conditions promoting the substitutional replacement. The increase in *J*_sc_ and FF behind the PCE jump from ∼18% to 19.6% reported by Wan *et al.*^91^ when introducing 5% Zn^2+^ was explained by means of DFT simulation. The calculation showed that the replacement of Ni^2+^ with Zn^2+^ reduces the nickel vacancy formation energy. Notably, a similar concept has been proposed by Kim *et al.*^39^ to explain the doping mechanism of Cu, whose most abundant oxidation state is Cu^2+^. However, Chen *et al.*^92^ revealed the presence of a minor fraction of Cu^+^ in NiO using XPS. The combined effects of increasing the number of nickel vacancies from Cu^2+^ along with lithium-type doping from Cu^+^ might explain the effectiveness of Cu doped NiO, also exploited in copper doped NiO nanoparticles in planar^106^ and mesoscopic configurations.^43^ When doped into NiO, cobalt exhibits a mixture of Co^2+^ and Co^3+^.^93^ Natu *et al.*,^107^ following the work of Radwanski *et al.*,^108^ proposed that Co^2+^ enhances the acceptor state density in NiO being able to accept an extra electron in its *t*_2g_ levels. The role of Co^3+^ is not clear. As a hypothesis, Co^3+^ might replace Ni^3+^ in the equilibria involving the nickel vacancies, *via* the following equation:5
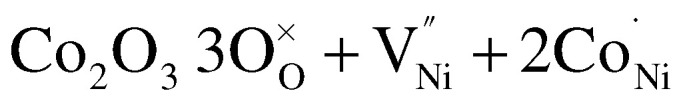


This is somehow similar to the mechanism proposed to explain the increase of p-type conductivity in Al^3+^ doped NiO.^109^ Besides several demonstrations of successful cobalt doping of NiO to increase the performances of PSCs,^95,110^ a particularly exciting study by Hou *et al.*^111^ reported a mixed amorphous Ni–Co oxide (with Ni : Co 1 : 1, even though the formation of NiCo_2_O_4_ cannot be discarded entirely), demonstrated as an efficient layer for 20% perovskite solar cells, paving the way for a broad family of mixed oxides as hole selective layers.

## 4. NiO/perovskite interface

The control of NiO/perovskite increases the degree of complexity for making highly efficient NiO-based perovskite solar cells. The chemistry of the NiO surface can be rather complex as one can notice from the several Ni peaks in XPS spectra.^112,113^ Different oxidation states (Ni^3+^ and Ni^2+^) due to defects or the presence of hydrates (NiOOH) and secondary phases (Ni_2_O_3_ or α/β –Ni(OH)_2_) are standard for low-temperature synthesis.^114–117^ These defects modify the energy levels, the carrier concentration and the mobility of the material, influencing the charge extraction, recombination rates, and the stability of the NiO/perovskite interface. The uneven surface also affects the growth of the perovskite layer, which amplifies the effect of the surface chemistry, requiring additional optimisation and complicating the comparison among different reports in the literature. The growth of the perovskite layer on any metal oxide often causes the decomposition of the organic cation at the interface, influencing the electronic properties and potentially increasing the recombination rates.^118–120^ In the specific case of NiO, this behaviour is affected by the defects present at the interface: as an example, the same perovskite deposition can lead to the formation of PbI_2_ when sputtered NiO is used while this phenomenon does not occur on solution-processed NiO.^121^ This excess is caused by the degradation of methylammonium at the interface with NiO, and to counteract this effect the perovskite would require either a post-treatment with additional cations or the use of an A-site cation excess in ink. Alternative deposition techniques such as the hot-casting method seem less prone to give rise to defects at the interface.^89^ These phenomena show that improvement in the energy alignment and reduction in defects during the perovskite growth can be achieved not only by modifying the synthesis of NiO but also by tailoring the perovskite deposition.

### 4.1 Charge dynamics at the NiO/perovskite interface

From a fundamental point of view, the NiO interface can extract holes in times down to the sub-picosecond scale, and the wide bandgap allows slowing down the recombination to the order of hundreds of picoseconds.^122^ For these reasons, the interface is not considered a limiting factor in the hole extraction when compared to PEDOT.^123^ However, the NiO interface can have a large number of defects. Defect engineering has been widely applied to improve the performance of NiO based devices, either by tuning the concentration of Ni^3+^ or by inducing the formation of additional NiOOH.^124–127^ Increasing the Ni^3+^ concentration can improve conductivity and charge transfer, but at the same time, it causes non-negligible parasitic absorption.^125,128^ The proper use of NiOOH also appear complex due to the very different energetic levels at the different crystalline surfaces.^129^ Extrinsic doping seems like a more promising approach because it allows tuning of the conductivity, work function and bandgap without the optical losses due to Ni^3+^. The presence of Ni^3+^ sites at the interface is also not required for the extraction of charges since there is evidence that holes are transferred to the Ni^2+^ sites.^130^

### 4.2 NiO/perovskite interface stability

**Fig. 3:**
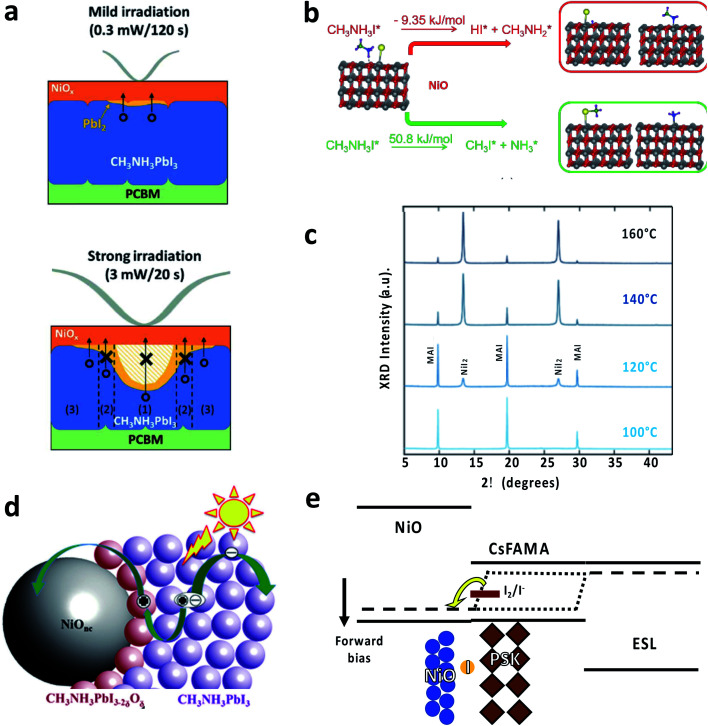
(a) The formation of PbI_2_ at NiO/CH_3_NH_3_PbI_3_ upon light irradiation and the effect on charge transport. Readapted with permission from ref. 131. Copyright 2018 Royal Society of Chemistry. (b) Reaction enthalpies for the decomposition of methylammonium iodide on the NiO surface. Reproduced with permission from ref. 132. Copyright 2020 American Chemical Society. (c) XRD spectra of NiO exposed to methylammonium iodide solution at increasing temperature showing the formation of NiI_2_. Readapted with permission from ref. 133. Copyright 2019 American Chemical Society. (d) Scheme for the formation of an oxygen-containing perovskite at the NiO interface. Reproduced with permission from ref. 134. Copyright 2016 John Wiley and Sons. (e) Scheme for redox chemistry at the NiO/perovskite interface. Readapted with permission from ref. 124. Copyright 2019 John Wiley and Sons.

Perovskite solar cells with NiO are usually considered to be very stable, with several reports showing high stability under light (with UV) and thermal stresses that would allow passing several IEC tests.^68,135–137^ The main reason is the structural stability of the NiO layer, preventing the layer of perovskite with the electrodes. Nevertheless, the NiO/perovskite interface can show some specific degradation mechanism that should be prevented to fabricate stable devices. In particular, the formation of NiI_2_ has been detected upon thermal degradation of the NiO/perovskite interface.^132,133^ The process is triggered by the large negative enthalpy of the NiO reaction with HI, arising from MAI (Fig. 3b).^132^ Under electrical bias, the interaction between NiO and iodide can result in redox reactions, which can explain the *J*–*V* hysteresis and impact the interface stability (Fig. 3e).^124^ Light soaking might be beneficial for improving the perovskite crystallinity^138^ or either induce interface degradation to NiI_2_ (Fig. 3c)^139^ or PbI_2_ (Fig. 3a).^131^ The formation of an oxygen-containing perovskite interphase (CH_3_NH_3_PbI_3−2*x*_O_*x*_, Fig. 3d) has been proposed as well, which can not only induce interfacial p-doping, but also open a new pathway towards degradation.^134^ It is worth noting that the reports on light soaking tests beyond 1000 hours employ inks with a stoichiometric composition of cations and anions, so preventing the formation of PbI_2_ could help to improve the durability of the interface.^135^ Other solutions are the use of hybrid interlayers such as magnesium acetate or ionic liquids such as 1-butyl-3-methylimidazolium tetrafluoroborate to enhance the interface between NiO and perovskite, with enhancements in both performance and stability.^124,135^ In particular, the former can improve the NiO interface electrical stability while the latter is useful to prevent the segregation of halides.

### 4.3 NiO functionalisation

Surface treatments with oxygen plasma or UV/O_3_ can be a valid approach to modify the surface of a metal oxide. Still, the results in perovskite solar cells are not consolidated and are, probably, strongly dependent on the starting conditions (the type of NiO and the perovskite synthesis).^124,140^ The functionalisation of the NiO surface appears more suitable to achieve reproducible improvements and to be less sensitive to the synthesis of NiO. A double layer is also often used in efficient OLEDs to differentiate the function of selective transport and charge injection, and a similar approach could be used here for hole extraction and electron blocking. The functionalisation of NiO can be achieved with inorganic,^46^ hybrid^124^ or organic compounds.^45,118,120,141–145^ We believe that this approach will be key to maximising the performance of PSCs with NiO. For instance, the introduction of a thin PTAA coating on NiO reduces the interface recombination^120^ and a similar effect has been observed with alternative polymers^146^ or by treating the NiO surface with different alkali halides: KCl,^46^ NaCl^98^ or CsBr.^147^ Another possibility is to graft self-assembled monolayers (SAMs) on the NiO surface, by bonding through amines,^118^ carboxylates^142^ and thiols^148^ with phosphonic acid also potentially effective. An organic interlayer, also exploiting the versatility due to the tunable molecular structure (especially in the case of SAMs), could tackle different issues such as the control of the surface chemistry, defect-free growth of perovskite, reduction of mechanical stress, and formation of covalent bonding. In this way, it will be possible to stabilise the interface, promote an appropriate growth of the perovskite and improve the *V*_OC_ of p–i–n solar cells to achieve efficiencies on par with n–i–p devices.

## 5. Broad application of NiO in perovskite photovoltaics

Perovskite photovoltaics is attractive for a broad range of applications. Low-temperature processing is compatible with flexible substrates. In combination with the excellent performances under indoor lighting,^149^ this could have a significant impact on the IoT (Internet of Things). Additionally, the low costs can be further abated by printing the counter electrode. Especially when considering consumer electronics and wearables the chance to go lead-free is of paramount importance.^150,151^

### 5.1 Flexible perovskite solar cells

**Fig. 4:**
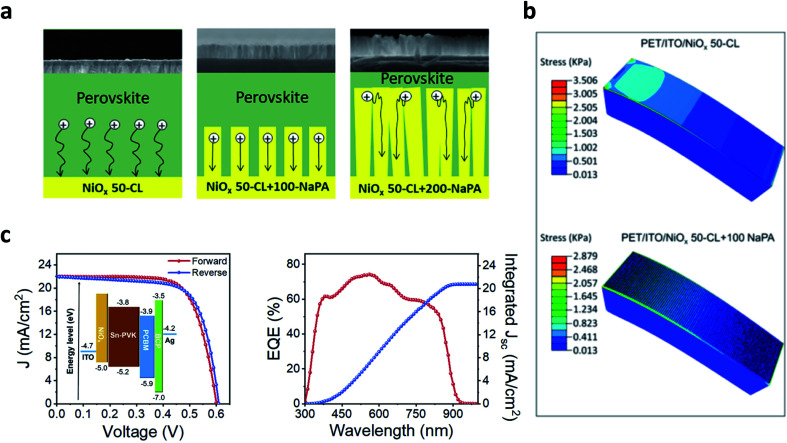
(a) Morphology and hole transfer mechanism for the NiO nanopillar array as discussed in ref. 152. (b) Finite element simulation showing the lower stress for the nanopillar array NiO layer, from ref. 152. (a) and (b) Reproduced with permission from ref. 152. Copyright 2019 American Chemical Society. (c) Device architecture, *J*–*V* curve and IPCE for a 10% efficient lead-free perovskite solar cell employing NiO as the hole selective layer. Reproduced with permission from ref. 153. Copyright 2018 Elsevier.

Plastic substrates limit the processing temperature to below 150 °C.^154^ This makes the NiO nanoparticle ink route the sole compatible solution process.^54,155^ Doping NiO nanoparticles in flexible PSCs boosts the efficiency from around 13% (undoped NiO)^54,155^ to above 16% with Cu : NiO^106^ and to 14.5% with Fe : NiO.^156^ A remarkable increase to 20% PCE has been achieved with the molecular dopant F2HCNQ.^38^ Excellent operational stabilities (>90% PCE after 500 h of MPPT) have been demonstrated combining a PCBM/ZnO electron selective layer and the NiO NP layer.^157^ A minor loss of efficiency after 1000 bending cycles at a radius of 5–8 mm was also demonstrated.^38,106,157^ 2-D materials might be particularly well suited for flexible electronics, thanks to their electrical and mechanical properties.^158^ In particular, graphene quantum dot (GQD) doping of the NiO NP layer has risen to prominence because it also allows us to explore different functionalisation of the 2D material.^159^ For instance, Zhang *et al.*^160^ showed an improvement in PCE from 14.6% to above 18% on a rigid device by chemically reducing the graphene oxide dopant with hydrazine or urea, also demonstrating a 14.1% PCE on a plastic substrate. Wang *et al.*^161^ investigated in detail the effect of the functionalising group, finding that hydroxy-functionalised GQDs induced a severe aggregation of the NiO nanoparticles within the precursor ink. At the same time, the amine functionalisation enabled the formation of a smooth and high-quality NiO layer leading to a PCE of 19.55% on a rigid substrate and of above 18% on PEN/ITO and maintaining 88% of PCE after 1000 bending cycles at a diameter of 10 mm. Further investigation on the effect of functionalised GQDs on the mechanical stability of flexible devices will be of high interest to consolidate this critical approach. An insightful analysis about the mechanical stability is provided by Cong *et al.*,^152^ who exploited e-beam evaporation with a glazing-angle atomic deposition layout (GLAD) to control the morphology of the NiO layer (Fig. 4a). The process is compatible with plastic substrates, and by varying the glancing angle it is possible to cast a compact layer (GA = 0°) or a nanopillar array (GA = 85°). The optimised nanopillar array increased the *J*_sc_ and the FF of the solar cells, and flexible devices with PCE above 17% were demonstrated. Moreover, finite element simulation showed that the presence of the nanopillar array on top of the compact layer reduced the mechanical stress upon bending (Fig. 4b), directly improving the mechanical stability of flexible perovskite solar cells.

### 5.2 Carbon-based printable perovskite solar cells

Triple mesoscopic stack perovskite solar cells with a printable carbon electrode (c-PSCs) are a subject of intense research due to their low cost and promising stability, especially when introducing 5-AVA into the perovskite composition.^162^ In c-PSCs, the perovskite is infiltrated through mp-TiO_2_ as an electron selective layer and a mesoporous Al_2_O_3_ or ZrO_2_ scaffold. The electrode is printed carbon, which guarantees higher stability concerning metal electrodes and partially acts as an encapsulating layer. The efficiency of HTM-free c-PSCs, which is the standard configuration, with the carbon electrode also functioning as a hole layer, is severely hindered by the low *V*_oc_ and FF. This drawback can be mitigated by introducing a p-type layer.^163^ Xu *et al.*^164^ observed a net increase in both *J*_sc_ (from 14.3 mA cm^−2^ to 21.3 mA cm^−2^) and *V*_oc_ (from 0.66 V to 0.76 V) when exploiting multiple mp-TiO_2_/mp-ZrO_2_/mp-NiO and a PCE approaching 15%. Improved energetic alignment between perovskite and a NiO containing carbon electrode might explain the PCE.^165–167^ Intensive research was after that dedicated to improving the efficiency of c-PSCs by modifying the NiO layer morphology,^168^ processing^169,170^ or doping.^171^ Still, more work is required to improve the PCE from about 15% substantially. However, efficiencies above 18% employing printable carbon electrodes have been demonstrated by adopting an n–i–p configuration with P3HT/graphene^172^ or CuSCN^173^ as a compact HSL on top of the perovskite, suggesting that future advancement in NiO processing on top of perovskites could enable stable and cheap carbon-based perovskite solar cells with minimal efficiency losses.

### 5.3 Lead-free perovskite solar cells

Lead-free perovskite solar cells with high efficiency are now only a matter of time, as confirmed by the certified efficiency of 12.4%.^174^ It is interesting to note that PEDOT:PSS is the most employed HSL for Sn-based PSCs.^175^ With lead halide perovskites, one of the driving forces to adopt NiO was the replacement of the relatively unstable PEDOT:PSS.^176,177^ The reasons behind the scarce application of NiO in Sn–PSCs are not known. The prime suspect is that the lower ionisation energy of PEDOT:PSS with respect to NiO might drive a better band alinement with the VB of tin halide perovskites, shallower than the Pb counterpart. Another possibility is that the NiOOH-rich surface might induce Sn^2+^ oxidation to Sn^4+^, degrading the interface. Ideally, these two issues might be tackled *via* interface engineering. The valence band of NiO can be shifted by anchoring molecular dipoles on the surface.^143^

Moreover, molecular monolayers have been proved excellent to protect perovskite surfaces and interfaces.^178^ We foresee that chemical or physical treatments of the NiO surface^179,180^ might also improve the optoelectronic quality of the NiO/Sn–perovskite interface. For instance, NaBH_4_ has been proved effective in reducing the NiO surface.^181^ This specific research topic is in its infancy, and still pioneering results push towards cautious optimism. A 3.31% efficient β-CsSnI_3_ PSC employing a sputtered-NiO/spin-coated NiO bi-layer was demonstrated by Wang *et al.*^182^ and an efficiency approaching 10% was obtained by Li *et al.*^183^ and Wang *et al.*^153^ (Fig. 4c).

## 6. Conclusion and outlook

The implementation of NiO as a hole selective layer in halide perovskite photovoltaics yields power conversion efficiencies above 20%, together with low cost, easy processing and long term stability. NiO nanoparticles will probably be the choice of material for flexible photovoltaics. Here, the synergy with 2D materials can be particularly fruitful by improving mechanical flexibility and reducing energy losses. When considering rigid substrates and tandem photovoltaics, processing by sputtering or spray pyrolysis might be the best choice for easy industrial adoption, with the TCO/NiO substrate processed in the same line. We have discussed in detail that a broad set of dopants can be introduced to enhance the conductivity of NiO or to improve the energy level alinement with halide perovskites. We have shown that each dopant acts in a different way depending on its size, valence and chemical identity. In our opinion a definitive choice of the best NiO dopant is not possible yet, especially considering the long term stability and the variety of perovskite formulations that can be used. Dopants can segregate at the NiO surface or diffuse inside the perovskite, and the doping stability has to be assessed in greater detail. In addition to that, we showed that pieces of evidence are accumulating stressing that NiO is particularly reactive for lead halide perovskites. Light, temperature and electrical bias can all trigger the NiO/perovskite reactivity. Thus, aiming at highly efficient and stable solar cells, the introduction of a passivation layer seems mandatory. Self-assembled monolayers can represent an ideal choice to passivate NiO defects, tuning the energy levels and promoting the growth of a high-quality perovskite film. An alternative might be the introduction of an ultra-thin layer (tunnelling layer) of inorganic insulating materials (*e.g.* MgO and Al_2_O_3_). Moreover, we believe that careful engineering of the NiO surface will enable efficient and stable lead-free perovskite solar cells.

## Conflicts of interest

There are no conflicts to declare.
